# Can we manipulate the ovary’s own metabolism to protect it from chemotherapy-induced damage?

**DOI:** 10.1038/s44321-024-00124-z

**Published:** 2024-09-03

**Authors:** Adomas Liugaila, Agnes Stefansdottir, Norah Spears

**Affiliations:** Biomedical Sciences, University of Edinburgh, Hugh Robson Building, George Square, Edinburgh, EH8 9XD UK

**Keywords:** Cancer, Metabolism, Urogenital System

## Abstract

Yes: A. Stefansdottir, A. Liugaila and N. Spears discuss the study from Ho et al, in this issue of *EMBO Mol. Med.*, that shows that nicotinamide mononucleotide supplementation ameliorates chemotherapy-induced female infertility without impairing treatment efficacy in breast cancer mouse models.

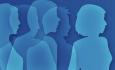

## Teaser

Some chemotherapy treatments induce female infertility through accelerated ovarian ageing, including due to nicotinamide adenine dinucleotide (NAD) depletion. Using various mouse models, Ho et al (2024) demonstrate that exposure to two such chemotherapy drugs, cisplatin or doxorubicin, deplete ovarian NAD, with levels restored by administrating the exogenous NAD precursor nicotinamide mononucleotide, ameliorating the drugs’ damaging effects on fertility.

Chemotherapy drugs have revolutionised the treatment of cancer in recent decades, with their continued development helping to dramatically increase patient survival for most cancers. Nonetheless, these drugs are undoubtedly a somewhat blunt instrument, with not only cancerous but also healthy cells exposed, inevitably leading to detrimental side-effects. Some of the better-known side-effects include hair loss and nausea, but treatment can also lead to more severe effects such as cardiomyopathy, a disease of the heart muscle. In addition, one tissue particularly vulnerable to toxic effects is the ovary, with infertility being a key non-survival-related concern for young female cancer patients.

The ovary contains the resting pool of ovarian primordial follicles (PFs), each consisting of an immature oocyte surrounded by a single layer of somatic granulosa cells, comprising a female’s lifetime store of oocytes. The pool of resting PFs is formed before birth, after which there is a gradual, slow release of follicles from that store, as they undergo growth initiation and develop towards the preovulatory stage, with normal physiology resulting in most dying through follicular atresia before they reach that point. This process continues throughout a female’s reproductive lifespan, but once too few PFs remain, she enters menopause, involving loss of fertility and other post-menopausal issues such as increased incidence of cardiovascular disease and osteoporosis. When younger pre-menopausal females, including pre-pubertal girls, are exposed to chemotherapy treatment, many will lose a marked proportion of their PFs, hastening ovarian ageing, leading to an earlier menopause with consequent negative impacts on health.

With steadily improving cancer treatments leading to increased survival rates, it is becoming more important than ever for cancer care not only to tackle the immediate issue of cancer itself but also to work towards ensuring a high quality of life after treatment. This includes the development of strategies to shield cancer survivors from off-target, long-term detrimental side-effects of chemotherapy treatment such as infertility. It is vital that such strategies achieve this aim without interfering with the efficacy of the cancer treatment itself. To date, strategies to address the risk of infertility have focussed on the use of fertility preservation techniques, namely cryopreserving oocytes, embryos or ovarian biopsies ahead of treatment. The best option, though, would be if we could protect the ovary against chemotherapy drug-induced damage from occurring in the first place, retaining the PF pool and hence preserving fertility whilst also improving long-term health outcomes (Alesi et al, 2023). The difficulty of developing an ovarian chemoprotectant is exacerbated by the fact that different types of chemotherapy drugs damage different cell types within the ovary, and through variable mechanisms of action. In addition, research is further complicated by an ongoing controversy as to whether the drugs lead directly to PF death and/or whether PFs are lost due to premature growth activation (Spears et al, 2019). Regardless, it is clear that chemotherapy treatment can result in a loss of PFs, and that any successful chemoprotectant needs to block that loss. A new manuscript published in this edition of EMBO Molecular Medicine explores one such potential protectant, through manipulation of the nicotinamide adenine dinucleotide (NAD) pathway (Ho et al, 2024). Using several different mouse models, the authors explore whether restoring the NAD+ metabolome can improve fertility following exposure to two commonly used chemotherapy drugs, the anthracycline doxorubicin (Dox) and the alkylating-like platinum-based agent cisplatin (Cis), without impacting anti-cancer effects.

The NAD pathway is a critical component of cell metabolism, energy production and viability, with NAD involved in both redox and non-redox reactions. Redox reactions are chemical reactions involving electron transfer, such as cellular respiration. NAD exists in two forms, oxidised (NAD+ ) and reduced (NADH), with the balance between the two determining the cell’s redox state. NAD is also involved in non-redox reactions, acting as a cofactor for enzymes such as CD38, involved in Ca^2+^ homoeostasis, inflammation and immune cell function, or poly(ADP-ribose) polymerases (PARPs), involved in a variety of cellular processes including that of early DNA repair responses: both use NAD+ in large amounts. Throughout the body, the activity of CD38 and PARPs increases with age, leading to a higher demand for NAD+ , but with this occurring alongside a depletion of NAD+ through ageing. Consequently, DNA repair rates decline, mutations and chromosomal abnormalities accumulate, ultimately leading to cellular dysfunction. The declining ovarian NAD+/NADH ratio is one reason why female fertility declines with increasing age: depleting NAD+ levels reduce the efficiency of oocyte DNA repair, diminishing the ovarian reserve and ultimately leading to ovarian ageing (Ahmed et al, 2024). This ageing process can be accelerated by many chemotherapy treatments, making the NAD pathway an attractive focus for an ovarian chemoprotectant.

Ho et al, manipulate the NAD pathway either by administering a metabolic precursor to NAD+ and NADH, nicotinamde mononucleotide (NMN), or through use of a transgenic mouse that overexpresses the NAD+ biosynthetic enzymes nicotinamide nucleotide adenylyltransferase 1/3. NAD treatment attenuates not only the chemotherapy drug-induced reduction in oocyte yield, but also long-term fertility. Perhaps surprisingly, this does not seem to be accompanied by protective effects on ovarian follicle number or health, although fewer unhealthy PFs are found in mice treated with Dox and NMN compared to those treated with Dox alone. The paper examines how NMN might act, analysing the NAD+ metabolome. Dox depleted levels of both NAD+ and also NADPH, a phosphorylated form of NADH involved in detoxification and metabolism of Dox, with levels of both restored by NMN administration. Hence, Ho et al, propose that NADPH levels fall as Dox is metabolised by NADPH-dependent enzymes, leading to the observed decline in NAD+ metabolome. During Dox treatment, large amounts of the drug need to be metabolised, requiring high levels of NADPH, potentially leading to a shortage of NAD+. Ho et al, noted that doxorubicinol, one of the NADPH-fuelled metabolites of Dox, causes cardiac dysfunction. To address this issue, they tested whether elevated NMN levels could induce cardiotoxicity: encouragingly, no such effects were found, a reassuring result regarding cardiac safety.

One vital aspect for any potential chemoprotectant is, of course, that it must not interfere with the efficacy of the cancer treatment. A key strength of Ho et al,’s study is that experiments were carried out on mice xenografted with a highly aggressive and invasive breast cancer cell line, receiving co-administration of NMN with Dox or Cis. Not only was there no interference of NMN with the tumour-suppressing activity of Dox or Cis in vivo, but NMN treatment alone reduced tumour volume, an unexpected and encouraging finding. In addition, in vitro experiments on six different cancer cell lines showed that NMN did not reduce the chemotherapy effectiveness, with that work also testing NMM alongside a range of different chemotherapy drugs. Together, this work provides promising evidence that administration of NMN alongside that of chemotherapeutics should not interfere with the cancer treatment on the tumour itself, in fact even providing additional evidence suggesting that it has the potential to augment efficacy.

Overall, this is an excellent study that uses an innovative approach to address an important concern in cancer care, that of oncofertility. A particular strength of the work is its combination of different techniques, using both pharmacological and transgenic strategies to manipulate the NAD(P)+ metabolome, as well as exploring its protective effects on several other off-target effects of concern, not just examining fertility. Chemotherapy drugs are usually administered in various combinations, and so future studies need to explore whether the protective effect of manipulating the NAD pathway applies to treatment with combinations of chemotherapy drugs, with further work also addressing whether treatment offers ovarian protection against other classes of chemotherapy drugs. Nonetheless, the paper points to the enhancement of the NAD(P)+ metabolome being a promising potential chemoprotective agent to ameliorate chemotherapy-induced female infertility without compromising anti-tumour activity.
